# Rehabilitation of Spinal Accessory Nerve Injury and Shoulder Dysfunction Following Neck Dissection for Head and Neck Cancer: A Narrative Review

**DOI:** 10.3390/jfmk11040382

**Published:** 2026-09-24

**Authors:** Rossella Calciano, Paola Emilia Ferrara, Alberto Cutaia, Elisabetta Lama, Anna Sanfilippo, Alessia Rabini, Alessandro de Sire, Gianpaolo Ronconi

**Affiliations:** 1 Department of Geriatrics, Orthopedics and Rheumatology, Fondazione Policlinico Universitario “Agostino Gemelli” IRCCS, L.go A. Gemelli 8, 00168 Rome, Italy; rossella.calciano@guest.policlinicogemelli.it (R.C.); paolaemilia.ferrara@policlinicogemelli.it (P.E.F.); gianpaolo.ronconi@policlinicogemelli.it (G.R.); 2 Department of Geriatric and Orthopedic Sciences, Università Cattolica del Sacro Cuore, L.go F. Vito 1, 00168 Rome, Italy; elisabetta.lama@guest.policlinicogemelli.it (E.L.); anna.sanfilippo@guest.policlinicogemelli.it (A.S.); 3 Dipartimento di Scienze Cardiovascolari, Policlinico A. Gemelli, L.go A. Gemelli 8, 00168 Rome, Italy; alessia.rabini@unicatt.it; 4 Department of Life Sciences, Health, and Health Professions, Link Campus University, Via del Casale di S. Pio V, 44, 00165 Rome, Italy; a.desire@unilink.it

**Keywords:** spinal accessory nerve injury, neck dissection, head and neck cancer, exercise rehabilitation, shoulder dysfunction, scapular dyskinesia, physiotherapy, functional recovery, quality of life

## Abstract

Spinal accessory nerve (SAN) injury is one of the most common causes of shoulder impairment after neck dissection for head and neck cancer. Dysfunction of trapezius muscle function is related to scapular biomechanics, and it results in pain, along with a reduction in range of motion, muscle weakness, and limitations in activities of daily living. Recently, exercise-based rehabilitation has been recognized as an important component of care. Evidence regarding the most effective rehabilitation strategies remains limited. The aim of this narrative review is to provide a comprehensive overview of SAN-related shoulder dysfunction following neck dissection for head and neck cancer, with particular emphasis on three main aspects: surgical strategies for preventing SAN injury, postoperative rehabilitation interventions for patients with established or suspected SAN-related dysfunction, and clinical and patient-reported outcome measures used to assess shoulder function and disability. A narrative review was conducted. Papers evaluating rehabilitation interventions for spinal accessory nerve injury after head and neck cancer surgery were included, and studies on rehabilitation interventions (manual therapy, strengthening, and supervised exercise) that are related to a reduction in pain and improvements in shoulder mobility, disability, and function were analyzed. Manual therapy enhanced cervical mobility and quality of life, strengthening improved short-term shoulder abduction, and supervised programs were more effective than home-based rehabilitation for pain and disability reduction. Targeted rehabilitation, including structured exercises and supervised physiotherapy, may improve shoulder mobility, reduce pain, and enhance functional recovery following spinal accessory nerve injury. Hospital-based supervised programs appear to offer greater short-term benefits. However, the limited evidence, small sample sizes, and heterogeneity in interventions prevent definitive recommendations. Further high-quality randomized trials with standardized protocols and long-term follow-up are needed.

## 1. Introduction

Shoulder dysfunction is one of the most prevalent conditions following neck dissection, with prevalence rates ranging from 30% to 80% depending on the type of surgery. Pain and functional dysfunction are present in up to 70% of patients, highlighting the limitations of current surgical techniques [1]. This type of impairment is related to an injury of the spinal accessory nerve (SAN), despite attention being paid to nerve preservation during surgery [1,2].

The reported prevalence of shoulder dysfunction after neck dissection varies widely according to the type and extent of neck dissection, spinal accessory nerve preservation, and assessment method. Adjuvant radiotherapy may also contribute to postoperative dysfunction, although evidence remains inconsistent. Therefore, prevalence estimates should be interpreted in light of this clinical and methodological heterogeneity [3,4].

Neck dissection is correlated with a lot of functional complications and limited mobility [5]. It occurs frequently after head and neck cancer, thus representing an important burden on global health. About 758,000 to 792,000 new cases and 424,000 deaths occur annually worldwide [6,7]. Diagnosis often occurs at 60–66 years old, with a male-to-female incidence ranging from 2:1 to 4:1 [8,9]. Surgical management includes cervical lymph node dissection, especially in the presence of lymph node metastases. Recently, improvements to surgical techniques have taken more conservative approaches, aiming for better nerve-sparing strategies, in order to reduce functional impairment.

The spinal accessory nerve (SAN) is the XI cranial nerve, and it provides motor innervation to the sternocleidomastoid and trapezius muscles, essential for head movement and stabilization of the shoulder [1]. The trapezius muscle is fundamental for maintaining normal scapulothoracic biomechanics and movement. This function is important for preserving physiological scapulohumeral rhythm and ensuring efficient upper limb movement during daily activities [10,11].

Damage to the SAN may result from direct surgical transection, traction injury, devascularization, or thermal injury during electrocautery [5,6,7,8,9,10,11,12]. Electromyographic studies have demonstrated denervation and neurogenic compromise of the SAN 4 to 6 months after the operation in all groups of patients who underwent neck dissection, with the most significant intraoperative injury occurring at anatomic levels IIa and IIb during dissection of the posterior cervical triangle [5,6,7,8,9,10,11,12]. The degree of dissection has an impact on the risk and severity of shoulder dysfunction, while adjuvant radiotherapy and chemotherapy have been identified as independent predictors of severe disability [1].

When SAN injury occurs, surgical reconstruction should be taken into consideration, particularly in cases of confirmed nerve transection or persistent functional deficit. Depending on the extent and location of the injury and the time elapsed since injury, reconstructive options include direct end-to-end nerve repair, interposition nerve grafting, and nerve transfers. Large clinical series have demonstrated favorable functional outcomes following both direct repair and nerve grafting of the SAN [13,14].

In selected cases in which primary repair or grafting is not feasible, nerve transfers have been described as an alternative reconstructive strategy [15,16].

Muscle or tendon transfer procedures, such as the Eden–Lange procedure, may provide an additional option for recovering shoulder function in patients with chronic and irreversible trapezius paralysis [17,18].

Therefore, recognition of SAN injury should not only prompt appropriate diagnosis and rehabilitation but also consideration of timely referral for peripheral nerve reconstruction when indicated.

The resulting shoulder dysfunction disrupts scapulohumeral rhythm and scapular stabilization. In particular, loss of trapezius activity forces compensatory recruitment of other scapular stabilizers such as the serratus anterior, rhomboids, and elevator scapulae, which are unable to reproduce normal trapezius function during shoulder elevation [10].

These mechanisms of compensation create inefficient shoulder movement, along with an increase in mechanical damage to the cartilage and joint [11]. Clinically, patients experience shoulder pain, weakness during arm elevation and an important reduction in range of motion. All these features are related to a reduction in the use of the shoulder during daily activities [19,20].

In the scientific literature, a series of studies highlight that 42% of patients after neck dissection experienced persistent spinal accessory nerve impairment with a reduction in functional outcomes [21]. Long-term follow-up studies of 5-year survivors confirm that decreased shoulder flexion and abduction remain associated with reduced quality of life [18]. These alterations may result in rotator cuff tendonitis, adhesive capsulitis, myofascial pain, chronic shoulder pain, muscular imbalance, and long-term functional disability if left untreated [1,2,3,4,5,6,7,8,9,10,11,12,13,14,15,16,17,18,19,20,21,22].

Accordingly, rehabilitation is crucial. The American Cancer Society (ACS) Head and Neck Cancer Survivorship Care Guideline recommends specific rehabilitation programs focused on strengthening of shoulder muscles, maintenance or improvement of range of motion, and optimization of function [1].

Strengthening of the scapular stabilizer, neuromuscular re-education, proprioceptive training, progressive range-of-motion exercises, manual therapy, soft tissue mobilization, and myofascial release are among the exercises that are provided in supervised clinical or home-based settings [10,11,12,13,14,15,16,17,18,19,20,21,22,23,24]. Among these, progressive resistance training (PRT) is supported by moderate evidence in systematic reviews and meta-analyses in improving shoulder pain, disability, and range of motion [25,26] Also, manual therapy combined with standard physiotherapy has also shown promise in reducing muscle stiffness and improving function, compared to standard physiotherapy alone [23].

Despite growing interest in this field, evidence gaps persist. The available literature is characterized by heterogeneity in study design, rehabilitation protocols, outcome measures, and follow-up durations [26,27,28]. In the context of the limited data on long-term functional recovery, the majority of research efforts have small patient samples and focus primarily on short-to-medium-term outcomes [27,28,29]. Furthermore, the optimal timing for initiating rehabilitation interventions remains unclear, as does the comparative effectiveness of different exercise modalities and dosing parameters [25,26,27,28].

It is also important to focus on prehabilitation programs and prevention. Prehabilitation is a process that takes place between diagnosis and surgery that consists of one or more preoperative interventions aimed at optimizing the patient’s physical and psychological condition before the surgical stress [30]. Recent systematic reviews and meta-analyses evaluated the effectiveness of prehabilitation programs in patients with head and neck cancer, including 31 studies utilizing quantitative analysis and 15 utilizing qualitative synthesis. These interventions are based on nutrition, exercise, and psychoeducation, and they are associated with improved outcomes after surgery. In particular, exercise-based prehabilitation is linked to improvements in the shoulder range of motion and movement as well as decreases in swallowing dysfunction and dysphagia. 

The aim of this narrative review was to give a thorough overview of shoulder dysfunction associated with spinal accessory nerve SAN injury following surgery for head and neck cancer, focusing on its assessment, prevention, and treatment. Specifically, we aimed to summarize the available evidence regarding the assessment of shoulder dysfunction, including clinical and patient-reported outcome measures; preventive and nerve-preserving surgical strategies; and postoperative rehabilitation and other therapeutic approaches aimed at improving shoulder function and reducing the functional effects of SAN injury.

## 2. Materials and Methods

All studies reported in PubMed, Scopus or Google Scholar and published in the last ten years are included in the study, after applying the specific search terms listed below.

The clinical question was structured according to the PICO framework [31]. The population (P) comprised patients with head and neck cancer undergoing neck dissection at risk of SAN-related shoulder dysfunction. The interventions (I) included preventive surgical strategies, postoperative rehabilitation, and therapeutic approaches aimed at preventing or improving shoulder dysfunction. Comparators (C) included standard care, no intervention, or alternative surgical and rehabilitative strategies. The outcomes (O) were shoulder function, range of motion, pain, muscle strength, quality of life, and functional disability.

For this narrative review, we included studies published as full-text articles in English, involving human participants, and published in their final form. The following topics were covered by eligible studies: (1) the assessment, prevention, or management of shoulder dysfunction following SAN injury; (2) postoperative rehabilitation strategies, including physiotherapy, exercise, and other rehabilitative interventions aimed at improving shoulder function; (3) preventive or nerve-preserving surgical approaches intended to reduce the incidence or severity of SAN injury and related shoulder dysfunction; and (4) the development, validation, reliability, or psychometric properties of clinical assessment tools and patient-reported outcome measures (PROMs) used to assess shoulder dysfunction in patients with SAN injury following head and neck cancer surgery. Studies were excluded if they were not related to head and neck cancer; did not involve SAN injury or SAN-related shoulder dysfunction; were unrelated to the assessment, prevention, rehabilitation, or management of shoulder dysfunction; involved non-human subjects; were not published in English; or were not available as full-text articles in their final peer-reviewed form. Conference abstracts, letters, editorials, comments, and duplicate were also not included.

A comprehensive search was conducted on PubMed, Scopus and Google Scholar using the following search strings with specific filters, as summarized in Table 1.

Pubmed ((“neck injuries”[MeSH Terms] OR (“neck”[All Fields] AND “injuries”[All Fields]) OR “neck injuries”[All Fields] OR (“neck”[All Fields] AND “injury”[All Fields]) OR “neck injury”[All Fields]) AND (“cancer s”[All Fields] OR “cancerated”[All Fields] OR “canceration”[All Fields] OR “cancerization”[All Fields] OR “cancerized”[All Fields] OR “cancerous”[All Fields] OR “neoplasms”[MeSH Terms] OR “neoplasms”[All Fields] OR “cancer”[All Fields] OR “cancers”[All Fields]) AND (“rehabilitant”[All Fields] OR “rehabilitants”[All Fields] OR “rehabilitate”[All Fields] OR “rehabilitated”[All Fields] OR “rehabilitates”[All Fields] OR “rehabilitating”[All Fields] OR “rehabilitation”[MeSH Terms] OR “rehabilitation”[All Fields] OR “rehabilitations”[All Fields] OR “rehabilitative”[All Fields] OR “rehabilitation”[MeSH Subheading] OR “rehabilitation s”[All Fields] OR “rehabilitational”[All Fields] OR “rehabilitator”[All Fields] OR “rehabilitators”[All Fields]) AND (“accessory nerve injuries”[MeSH Terms] OR (“accessory”[All Fields] AND “nerve”[All Fields] AND “injuries”[All Fields]) OR “accessory nerve injuries”[All Fields] OR (“accessory”[All Fields] AND “nerve”[All Fields] AND “injury”[All Fields]) OR “accessory nerve injury”[All Fields])) AND ((y_10)) AND (humans[Filter]) AND (english[Filter])).

Google Scholar (spinal accessory nerve) AND (injury) (shoulder dysfunction) AND (neck dissection) AND (neck cancer) AND (rehabilitation) AND (english) AND (human).

Scopus TITLE—ABS—KEY spinal accessory nerve injury AND rehabilitation.

Reviews, case reports, duplicates, and meta-analyses were excluded manually.

All articles with “case report” in the title were excluded in the first step of article selection. For all articles finally eligible for this study, full-text articles were downloaded from the respective journals’ websites. The one study found on Scopus was excluded, as it was a study protocol and did not report results. Those studies finally eligible for the study were reviewed by both the first author (RC) and senior author (PEF). Any disagreements between the two reviewers regarding study selection, eligibility, or data interpretation were resolved through discussion and consensus; when consensus could not be reached, a third reviewer (AC) was consulted to make the final decision.

No additional analyses were performed. After the removal of duplicates and review by title and abstract six full-text articles were assessed for eligibility and were included in descriptive synthesis. The studies included are 3 randomized control trials (RCTs).

Data extracted from the selected studies were the inclusion criteria of each study, the number of participants, group data as to males and females, characteristics of the study group and control group, outcomes and main results.

All these are summarized in Table 2.

## 3. Results

The six studies that make up this narrative review are divided into different topics: prevention of SAN injury during neck dissection, postoperative rehabilitation, and assessment of shoulder dysfunction and related outcomes. Regarding prevention, RCTs investigated strategies aimed at preserving SAN function during surgery. Dziegielewski et al. [5] evaluated the preservation of level IIb during selective neck dissection, whereas Barber et al. [32] examined intraoperative brief electrical stimulation of the SAN. Regarding postoperative rehabilitation, three clinical studies evaluated rehabilitation interventions. Simsek et al. [23] investigated manual therapy combined with standard physiotherapy, McGarvey et al. [29] assessed early intensive physiotherapy that included scapular strengthening and progressive exercises, and Do et al. [24] compared hospital-based supervised exercise with a home-based exercise program. Regarding outcome assessment, Eden et al. [33] investigated the psychometric properties of shoulder-specific patient-reported outcome measures in patients after neck dissection, and found that the Neck Dissection Impairment Index had the best psychometric qualities of all the instruments evaluated. Overall, the six included studies comprised a total of 347 patients.

The findings were synthesized descriptively according to the three thematic domains. Rehabilitation interventions were evaluated in three clinical trials and included manual therapy combined with standard physiotherapy, early intensive physiotherapy with scapular strengthening and progressive resistance exercises, and hospital-based supervised exercise compared with home-based exercise programs [23,24,25,26,27,28,29]. Surgical strategies for SAN preservation were assessed by Dziegielewski et al. [5], who investigated omission of level IIb dissection, and by Barber et al. [32], who evaluated intraoperative brief electrical stimulation (BES) of the SAN. Eden et al. [33], instead, analyzed the psychometric assessment of shoulder function using a variety of instruments. Follow-up duration is various. Eden et al. [33] has assessment between 2 weeks and 18 months after neck dissection. Simsek et al. [23] assessed outcomes after a 6-week intervention; McGarvey et al. [29] evaluated outcomes at 3, 6, and 12 months; and Do et al. [24] assessed outcomes after 4 weeks of intervention. There is no follow-up in Dziegielewski et al. [5].

Also, outcome measures were heterogeneous. Functional outcomes included cervical and shoulder active range of motion (AROM) [23,24], shoulder strength and Constant–Murley Shoulder Score [32], and the Neck Dissection Impairment Index (NDII) [5,6,7,8,9,10,11,12,13,14,15,16,17,18,19,20,21,22,23,24,25,26,27,28,29,30,31,32,33,34].

Pain and disability were assessed using the Shoulder Pain and Disability Index (SPADI) [23,24,25,26,27,28,29], the Neck Disability Index (NDI) and a pain numerical rating scale (NRS) [24].

Neurophysiological evaluation and biomechanical outcomes included SAN conduction studies, compound muscle action potential (CMAP), and muscle mechanical properties assessed by shear wave elastography (SWE). Quality of life was evaluated using FACT-H&N [23] and EORTC QLQ-C30/EORTC QLQ-H&N35 [24]. Eden et al. [33] compared NDII, SPADI, QuickDASH, and DASH using Rasch analysis, identifying NDII as the PROM with the strongest psychometric properties for evaluating shoulder dysfunction after neck dissection.

Simsek et al. [23] demonstrated that manual therapy combined with standard physiotherapy produced significantly greater improvements than standard physiotherapy alone. The intervention group showed greater reductions in muscle stiffness measured by shear modulus of the sternocleidomastoid and upper trapezius muscles (*p* = 0.001) and improvements in cervical AROM (lateral flexion, extension, and rotation; *p* = 0.001), shoulder abduction (*p* < 0.05), and quality of life (FACT-H&N; *p* = 0.001). Both groupsdemonstrated significant improvements in pain (IG *p* = 0.004; CG *p* = 0.003) and SPADI scores (IG *p* = 0.005; CG *p* = 0.003), without significant between-group differences. McGarvey et al. [29] reported a statistically significant improvement in shoulder abduction in the early intensive physiotherapy group at 3 months, with a mean between-group difference of 26.6° (95% CI: 7.3–46°). However, no significant between-group differences were observed at 6 or 12 months for shoulder range of motion, SPADI, NDII, or GPES scores. These results imply that the advantages of intensive physiotherapy were primarily evident in the short term and were limited to a specific functional outcome. Although both groups demonstrated improvements over time, the available evidence does not demonstrate a sustained superiority of intensive physiotherapy over the usual care at 6–12 months. Therefore, these results should be interpreted as evidence of an early functional benefit rather than definitive evidence of a persistent treatment advantage. Dziegielewski et al. [5] showed that preservation of level IIb during selective neck dissection was associated with improved postoperative shoulder function, higher NDII scores, and reduced SAN impairment. Barber et al. [32] demonstrated that intraoperative BES of the SAN improved postoperative shoulder function and electrophysiological outcomes, particularly in patients having level IIb or level V neck dissection. Eden et al. [33] demonstrated the strongest psychometric properties and identified the most appropriate PROMs for assessing shoulder dysfunction after neck dissection.

## 4. Discussion

This narrative review analyzed the current available evidence on rehabilitation strategies and outcome measures for SAN dysfunction following neck dissection in patients treated for head and neck cancer. Only six studies met the inclusion criteria and they showed a lot of variability.

The findings of this narrative review should be interpreted according to the different objectives of the included studies. Preventive surgical interventions, such as preservation of level IIb and intraoperative brief electrical stimulation of the SAN, primarily aim to reduce the risk of postoperative nerve dysfunction rather than treat an established SAN injury. Therefore, it is important to separate these approaches from postoperative rehabilitation interventions, which aim to enhance shoulder mobility, strength, pain, and functional performance in patients with shoulder dysfunction. In addition, studies focusing on patient-reported outcome measures offer crucial information on the assessment of disability and the evaluation of treatment-related improvements, but they do not constitute therapeutic interventions. This distinction is essential for an appropriate interpretation of the available evidence and for avoiding direct comparisons between preventive, rehabilitative, and assessment-based approaches.

Preservation of level IIb and intraoperative brief electrical stimulation of the SAN are primarily intended to prevent or reduce nerve dysfunction during neck dissection, whereas postoperative physiotherapy and exercise-based interventions aim to improve functional recovery in patients with established or suspected SAN-related shoulder dysfunction. Although both approaches may contribute to a comprehensive continuum of care, the findings of preventive surgical studies cannot be interpreted as evidence of the efficacy of subsequent rehabilitation. Accordingly, the effects of these interventions should be evaluated independently, and no conclusions regarding the effectiveness of postoperative rehabilitation should be based on their combined effects.

Previous systematic reviews have supported physiotherapy and progressive resistance training for improving shoulder pain and disability after treatment for head and neck cancer [17,18]. Harris et al. [27] similarly concluded that most prospective studies demonstrated a beneficial effect of physiotherapy on postoperative shoulder function. The current review tries to improve this evidence by incorporating more recent studies investigating manual therapy as an adjunct to conventional physiotherapy and preventive intraoperative interventions aimed at preserving SAN function.

An important aspect of the current evidence is that the included studies focused on different stages of the patient’s management. Two RCTs highlighted the need for prevention of the SAN dysfunction during surgery; this is possible through the evaluation of the preservation of level IIb lymph node dissection and intraoperative brief electrical stimulation (BES) of the nerve [5,6,7,8,9,10,11,12,13,14,15,16,17,18,19,20,21,22,23,24,25,26,27,28,29,30,31,32,34]; One RCT examined the necessity of postoperative care, considering a combination of manual therapy and conventional physiotherapy. The last study evaluated functional aspects of shoulder dysfunction after this type of injury through specific tools. Our findings support the importance of a comprehensive continuum of care; however, preventive surgical strategies and postoperative rehabilitation address different clinical goals and should not be considered interchangeable.

Although the evidence remains limited to a single randomized trial, the findings of Simsek et al. [23] support the hypothesis that manual therapy may improve postoperative recovery by addressing soft tissue restrictions, enhancing cervical mobility and range of motion, and facilitating shoulder function and movement. Similar observations have been reported in the study by Castro-Martín et al. [35] that studied how myofascial induction therapy reduced cervical and shoulder pain and improved cervical mobility and muscle function following cancer treatment in head and neck cancer survivors. Similarly, a paper by McMilan et al. has shown that manual therapy targeting radiation-associated cervical fibrosis is both safe and linked to significant improvements in cervical movement [36]. These studies speculate about the importance of rehabilitation programs; however, they were not specifically designed to address patients with SAN dysfunction after neck dissection. 

The findings regarding preventive surgical strategies analyze the importance of preserving SAN integrity whenever it is oncologically possible. Dziegielewski et al. [5] reported increased postoperative shoulder function following preservation of level IIb during neck dissection, whereas Barber et al. [32] found that intraoperative BES was associated with better postoperative nerve function and shoulder performance.

These studies try to suggest that reducing the intraoperative risk of nerve injuries may reduce the postoperative impairment and dysfunction.

This concept is in line with a previous paper that highlighted the value of multidisciplinary management and early intervention to minimize disability after surgical SAN injury [37]. Rehabilitation is still crucial for this condition, but prevention is also important in order to give a continuum of treatment. The current evidence aligns with existing clinical recommendations for survivors of head and neck cancer. The American Cancer Society Head and Neck Cancer Survivorship Care Guideline recommends rehabilitation as an integral component of survivorship care, with Level IA evidence for improving shoulder function and daily activities [1].

A systematic review by Ekici et al. suggests that exercise-based interventions improve shoulder mobility, swallowing function, dysphagia, and other symptoms related to head and neck cancer, supporting the integration of structured rehabilitation into routinary and clinical postoperative management [38].

Although the available evidence has focused on general functional recovery rather than shoulder-specific outcomes, prehabilitation is a potentially useful approach for the perioperative management of patients with head and neck cancer. However, none of the six studies included in this narrative review directly evaluated the efficacy of a shoulder-specific prehabilitation program before neck dissection. The potential role of prehabilitation in preventing postoperative SAN-related shoulder dysfunction cannot be established from the findings of the present review [34]. Further prospective studies are needed to investigate whether individualized preoperative programs targeting shoulder mobility, scapular control, and upper-limb function can reduce postoperative impairment and improve functional recovery.

The significant variation in outcome measures in the research must also be highlighted. The papers combined clinician-reported functional scales, objective neurophysiological assessments, biomechanical muscle tools and patient-reported questionnaires. Such variability very limits comparisons between studies and complicates evidence synthesis. In this context, the observational study by Eden et al. [33] provides valuable information by comparing the psychometric properties of four commonly used PROMs. The Neck Dissection Impairment Index (NDII) seems to be the most suitable and proven tool for assessing shoulder dysfunction after neck dissection.

### 4.1. Limitations

There are a few restrictions to be aware of. First of all, the study included only six papers with small sample sizes and limited results that weakened the generalizability of the evidence. Second, there was a variation in the kinds of interventions and outcomes. The small number of randomized studies and the lack of a meta-analysis precluded a quantitative synthesis and limited the strength of the current conclusions. Furthermore, it is essential to underline the rehabilitation protocols and the different associated components, such as intensity, frequency, and duration. These methodological differences make it difficult to determine which specific rehabilitation or preventive program provides the greatest clinical benefit or whether certain patient subgroups respond differently to treatment.

### 4.2. Perspectives

Future research should prioritize larger sample sizes, multicenter randomized controlled trials using standardized rehabilitation protocols, and common outcome measures. Studies should separately investigate preventive surgical strategies, postoperative rehabilitation interventions, and shoulder-specific prehabilitation programs. Accordingly, adoption of standardized, validated outcome measures such as the NDII may consequently enhance future rehabilitation research and make cross-trial comparisons easier. Longer follow-up periods are needed to determine whether early functional improvements are maintained over time and to identify predictors of persistent disability. Future studies should also take into account the influences of significant clinical variables, like radiotherapy, chemotherapy, baseline shoulder function, differences between sex and age, and other previous musculoskeletal conditions. This analysis should facilitate the development of individualized rehabilitation pathways and strategies for this type of patient. The future literature should also underline the importance of prehabilitation programs that could help to reduce shoulder dysfunction and other complications after neck dissection and nerve injuries.

## 5. Conclusions

Spinal accessory nerve injury remains one of the most important causes of shoulder dysfunction after head and neck cancer surgery. The current evidence has reported the importance of both rehabilitation and preventive interventions, as demonstrated by this narrative review. These measures may help to improve shoulder mobility, reduce pain, and help functional recovery. However, conclusive recommendations are impossible due to the paucity of studies, small sample sizes, and variations in interventions and results. Further high-quality randomized trials are needed to develop standardized rehabilitation protocols, evaluate their long-term effects, and identify the predictors of disability and dysfunction.

## Figures and Tables

**Table 1:** Search strategy.

Database	Search Strategy	Filters Applied	Records Retrieved	Studies Included
PubMed	((“neck injuries”[MeSH Terms] OR “neck injury”) AND (“neoplasms”[MeSH Terms] OR cancer) AND (“rehabilitation”[MeSH Terms] OR rehabilitation) AND (“accessory nerve injuries”[MeSH Terms] OR “spinal accessory nerve injury”))	Last 10 years; Humans; English; Free full text; Free text	5	Simsek et al. [23]; Dziegielewski et al. [5]; Barber et al. [32]; Do et al. [24]; McGarvey et al. [29].
Google Scholar	(spinal accessory nerve) AND injury AND shoulder dysfunction AND neck dissection AND neck cancer AND rehabilitation	Last 10 years; English; Humans	140	Eden et al. [33].
Scopus	TITLE-ABS-KEY (“spinal accessory nerve injury” AND rehabilitation)	Last 10 years; English; Humans; Article; Journal; Open Access; Final publication	12	-

**Table 2:** Characteristics of the studies.

Author (Years)	Design of the Study	Pathology	Topic	Study Group	Control Group	Outcome Mesaures	Follow-Up	Results
Simsek 2025 [23]	Prospective double-blind RCT	Muscular stiffness	Postoperative rehabilitation	Manual therapy (myofascial and soft tissue techniques, scapular mobilization) + standard physiotherapy for 6 weeks N patients: 10 M/F: 4/6 Mean age (SD): 53.1 (17.09)	Standard physiotherapy (ROM, strengthening, stretching, scar massage, education) for 6 weeks N patients: 11 M/F: 6/5 Mean age (SD): 52 (16.04)	SWE muscle properties, cervical and shoulder AROM, SPADI, FACT-H&N	T0: baseline T1: 6 weeks	Greater reduction in muscle stiffness (*p* = 0.001), greater improvement in cervical ROM (*p* = 0.001), shoulder abduction (*p* < 0.05), and QoL (*p* = 0.001) in IG. Pain and SPADI improved in both groups, without between-group differences.
Dziegielewski 2020 [5]	Double-blind RCT	Shoulder symptoms	Prevention of SAN injury	Selective neck dissection with preservation of level IIb N patients: 15 M/F: 14/1 Mean age: 59.8	Conventional selective neck dissection including level IIb N patients: 15 M/F: 12/3 Mean age: 64.3	NDII, shoulder strength, shoulder ROM, SAN conduction	T0: baseline T1: 6 months	Preservation of level IIb significantly improved shoulder function, NDII scores, shoulder strength, ROM, and reduced SAN dysfunction, compared with standard dissection.
Barber 2018 [32]	Double-blind RCT	Shoulder symptoms	Prevention of SAN injury	Intraoperative brief electrical stimulation (BES) of the SAN N patients: 27 M/F: 23/5 Mean age: 57.8	No electrical stimulation N patients: 27 M/F: 21/6 Mean age: 57.9	Constant–Murley Shoulder Score, NDII, CMAP	T0: baseline T1: 12 months	BES significantly preserved postoperative shoulder function and electrophysiological nerve function at 12 months, particularly after level IIb + V dissection.
Eden 2021 [33]	Cross-sectional observational study	Shoulder symptoms	Assessment of shoulder dysfunction and outcomes	Psychometric evaluation of shoulder-specific PROMs N patients: 143 M/F: 113/30 Mean age: 62.67	-	Reliability, construct validity, measurement properties (Rasch analysis)	-	NDII demonstrated the strongest psychometric properties and was identified as the most appropriate PROM for assessing shoulder dysfunction after neck dissection.
McGarvey 2015 [29]	Multicenter RCT	Shoulder symptoms	Postoperative rehabilitation	Early intensive physiotherapy: progressive scapular strengthening, ROM, stretching and home program for 12 weeks N patients: 32 M/F: 24/8 Mean age: 57.4	Usual care (self-directed exercises, posture advice, scar massage) N patients: 27 M/F: 19/8 Mean age: 59.6	Shoulder ROM, SPADI, NDII, GPES	T0: baseline T1: 3 months T2: 6 months T3: 12 months	Shoulder abduction significantly greater at 3 months (mean difference 26.6°, 95% CI 7.3–46), but no between-group differences at 6 or 12 months. Both groups improved over time in ROM, SPADI, NDII and GPES.
Do 2018 [24]	Controlled, non-randomized clinical trial	Shoulder symptoms	Postoperative rehabilitation	Hospital-based supervised exercise (ROM, massage, stretching, progressive resistance) 40 min/session, 3×/week for 4 weeks N patients: 20 M/F: 9/11 Mean age: 56.2	Home-based exercise program with identical exercises N patients: 20 M/F: 8/12 Mean age: 49.6	EORTC QLQ-C30, EORTC QLQ-H&N, NDI, neck and shoulder ROM, pain (NRS)	T0: baseline T1: 1 month	Supervised exercise significantly improved NDI (*p* = 0.006), neck extension (*p* = 0.007), neck rotation (*p* = 0.001), and pain (*p* = 0.001). Both groups improved shoulder ROM and QoL, without significant between-group differences.

Abbreviation: RCT: randomized controlled trial; IG, intervention group; SAN, spinal accessory nerve; ROM, range of motion; AROM, active range of motion; SWE, shear wave elastography; SPADI, Shoulder Pain and Disability Index; FACT-H&N, Functional Assessment of Cancer Therapy–Head and Neck; NDII, Neck Dissection Impairment Index; CMAP, compound muscle action potential; PROMs, patient-reported outcome measures; GPES, Global Perceived Effect Scale; NDI, Neck Disability Index; EORTC QLQ-C30, European Organisation for Research and Treatment of Cancer Quality of Life Questionnaire Core 30; NRS, numeric rating scale; QoL, quality of life.

## Data Availability

No new data were created or analyzed in this study.

## References

[1] Cohen E.E., LaMonte S.J., Erb N.L., Beckman K.L., Sadeghi N., Hutcheson K.A., Stubblefield M.D., Abbott D.M., Fisher P.S., Stein K.D. (2016). American Cancer Society Head and Neck Cancer Survivorship Care Guideline. CA Cancer J. Clin..

[2] Gane E.M., Michaleff Z.A., Cottrell M.A., McPhail S.M., Hatton A.L., Panizza B.J., O’Leary S.P. (2017). Prevalence, incidence, and risk factors for shoulder and neck dysfunction after neck dissection: A systematic review. Eur. J. Surg. Oncol..

[3] Mohamad N., de Oliveira-Souza A.I.S., Ntoukas S.M., de Castro-Carletti E.M., Munajat M., Dennett L., Courneya K.S., Armijo-Olivo S., McNeely M.L. (2025). Musculoskeletal Impairments and Dysfunction in Individuals with Head and Neck Cancer Following Surgery with Neck Dissection—A Systematic Review. Life.

[4] Larsen M.H., Lorenzen M.M., Bakholdt V., Sørensen J.A. (2020). The prevalence of nerve injuries following neck dissections—A systematic review and meta-analysis. Dan. Med. J..

[5] Dziegielewski P.T., McNeely M.L., Ashworth N., O’connell D.A., Barber B., Courneya K.S., Debenham B.J., Seikaly H. (2020). 2b or Not 2b? Shoulder Function After Level 2b Neck Dissection: A Double-Blind Randomized Controlled Clinical Trial. Cancer.

[6] Deng M., Lin Y., Yan L., Fei Z., Chen C., Ding J. (2025). Global Burden of Head and Neck Cancer from 1990 to 2021: A Comprehensive Analysis and Projections to 2030 Based on the Global Burden of Disease Study 2021. PLoS ONE.

[7] Rumgay H., Colombet M., Ramos da Cunha A., Filho A.M., Warnakulasuriya S., Conway D.I., Chaturvedi A., Virani S., Lauby-Secretan B., Carvalho A.L. (2026). Global Incidence of Lip, Oral Cavity, and Pharyngeal Cancers by Subsite in 2022. CA Cancer J. Clin..

[8] Mody M.D., Rocco J.W., Yom S.S., Haddad R.I., Saba N.F. (2021). Head and Neck Cancer. Lancet.

[9] Dunn L.A., Ho A.L., Pfister D.G. (2025). Head and Neck Cancer. JAMA.

[10] McGarvey A.C., Osmotherly P.G., Hoffman G.R., Chiarelli P.E. (2013). Scapular Muscle Exercises Following Neck Dissection Surgery for Head and Neck Cancer: A Comparative Electromyographic Study. Phys. Ther..

[11] Contemori S., Panichi R., Biscarini A. (2019). Effects of Scapular Retraction/Protraction Position and Scapular Elevation on Shoulder Girdle Muscle Activity During Glenohumeral Abduction. Hum. Mov. Sci..

[12] Karthikeyan S., Joshi D., Pathak A., Pandey M. (2025). Impairment of Neck and Shoulder Function After Neck Dissection: A Comparative Study of Goniometry, EMG and Paper Pencil Tool in a Randomized Controlled Trial of Level IIb Preserving Versus Conventional Neck Dissection. World J. Surg. Oncol..

[13] Kim D.H., Cho Y.J., Tiel R.L., Kline D.G. (2003). Surgical outcomes of 111 spinal accessory nerve injuries. Neurosurgery.

[14] Park S.H., Esquenazi Y., Kline D.G., Kim D.H. (2015). Surgical outcomes of 156 spinal accessory nerve injuries caused by lymph node biopsy procedures. J. Neurosurg. Spine.

[15] Novak C.B., Mackinnon S.E. (2004). Treatment of a proximal accessory nerve injury with nerve transfer. Laryngoscope.

[16] Maldonado A.A., Spinner R.J. (2017). Lateral pectoral nerve transfer for spinal accessory nerve injury. J. Neurosurg. Spine.

[17] Teboul F., Bizot P., Kakkar R., Sedel L. (2005). Surgical management of trapezius palsy. J. Bone Jt. Surg..

[18] O’Driscoll J., Minarro J.C., Sanchez-Sotelo J. (2024). Paralysis of the trapezius muscle: Evaluation and surgical management. JSES Rev. Rep. Tech..

[19] Ortiz-Comino L., Fernández-Lao C., Speksnijder C.M., Lozano-Lozano M., Tovar-Martín I., Arroyo-Morales M., Martín-Martín L. (2020). Upper Body Motor Function and Swallowing Impairments and Its Association in Survivors of Head and Neck Cancer: A Cross-Sectional Study. PLoS ONE.

[20] Eickmeyer S.M., Walczak C.K., Myers K.B., Lindstrom D.R., Layde P., Campbell B.H. (2014). Quality of Life, Shoulder Range of Motion, and Spinal Accessory Nerve Status in 5-Year Survivors of Head and Neck Cancer. PM&R.

[21] Zhang H., Wang F., Gu M., Wan Q., Guo R., Duan L., Tian M. (2025). Quantifying Neck Disability After Neck Dissection in HNSCC: A Prospective Study of Patient-Reported and Objective Functional Outcomes. Head Neck.

[22] Huang Y.C., Lee Y.Y., Tso H.H., Chen P.-C., Chen Y.-C., Chien C.-Y., Chung Y.-J., Leong C.-P. (2019). The Sonography and Physical Findings on Shoulder After Selective Neck Dissection in Patients with Head and Neck Cancer: A Pilot Study. BioMed Res. Int..

[23] Simsek F., Naci B., Kilicoglu M.B., Alkan Z., Topcuoglu O.M., Gormez A., Hafiz G., Okyar A.F. (2025). Can Manual Therapy Alter Muscle Stiffness in Patients with Spinal Accessory Nerve Injury?. Otolaryngol.–Head Neck Surg..

[24] Do J.H., Yoon I.J., Cho Y.K., Ahn J.S., Kim J.K., Jeon J. (2018). Comparison of Hospital Based and Home Based Exercise on Quality of Life, and Neck and Shoulder Function in Patients with Spinal Accessory Nerve Injury After Head and Neck Cancer Surgery. Oral Oncol..

[25] Almeida K.A.M., Rocha A.P., Carvas N., Pinto A.C.P.N. (2020). Rehabilitation Interventions for Shoulder Dysfunction in Patients with Head and Neck Cancer: Systematic Review and Meta-Analysis. Phys. Ther..

[26] Carvalho A.P., Vital F.M., Soares B.G. (2012). Exercise Interventions for Shoulder Dysfunction in Patients Treated for Head and Neck Cancer. Cochrane Database Syst. Rev..

[27] Harris A.S. (2020). Do Patients Benefit From Physiotherapy for Shoulder Dysfunction Following Neck Dissection? A Systematic Review. J. Laryngol. Otol..

[28] McGarvey A.C., Chiarelli P.E., Osmotherly P.G., Hoffman G.R. (2011). Physiotherapy for Accessory Nerve Shoulder Dysfunction Following Neck Dissection Surgery: A Literature Review. Head Neck.

[29] McGarvey A.C., Hoffman G.R., Osmotherly P.G., Chiarelli P.E. (2015). Maximizing Shoulder Function After Accessory Nerve Injury and Neck Dissection Surgery: A Multicenter Randomized Controlled Trial. Head Neck.

[30] Carli F., Scheede-Bergdahl C. (2015). Prehabilitation to enhance perioperative care. Anesthesiol. Clin..

[31] Huang X., Lin J., Demner-Fushman D. (2006). Evaluation of PICO as a knowledge representation for clinical questions. AMIA Annu. Symp. Proc..

[32] Barber B., Seikaly H., Ming Chan K., Beaudry R., Rychlik S., Olson J., Curran M., Dziegielewski P., Biron V., Harris J. (2018). Intraoperative Brief Electrical Stimulation of the Spinal Accessory Nerve (BEST SPIN) for Prevention of Shoulder Dysfunction After Oncologic Neck Dissection: A Double-Blinded, Randomized Controlled Trial. J. Otolaryngol.–Head Neck Surg..

[33] Eden M.M., Kunze K.L., Galantino M.L., Kolber M.J., Cheng M.S. (2021). Shoulder-Specific Patient-Reported Outcome Measures for Use in Patients with Head and Neck Cancer: An Assessment of Reliability, Construct Validity, and Overall Appropriateness of Test Score Interpretation Using Rasch Analysis. Phys. Ther..

[34] Seth I., Bulloch G., Qin K.R., Xie Y., Sebastian B., Liew H., Rozen W.M., Lee C.H.A. (2024). Pre-Rehabilitation Interventions for Patients with Head and Neck Cancers: A Systematic Review and Meta-Analysis. Head Neck.

[35] Castro-Martín E., Galiano-Castillo N., Fernández-Lao C., Ortiz-Comino L., Postigo-Martin P., Arroyo-Morales M. (2021). Myofascial Induction Therapy Improves the Sequelae of Medical Treatment in Head and Neck Cancer Survivors: A Single-Blind, Placebo-Controlled, Randomized Cross-Over Study. J. Clin. Med..

[36] McMillan H., Warneke C.L., Buoy S., Porsche C., Savage K., Lai S.Y., Fuller C.D., Hutcheson K.A. (2025). Manual Therapy for Fibrosis-Related Late Effect Dysphagia in Head and Neck Cancer Survivors: The MANTLE Nonrandomized Clinical Trial. JAMA Otolaryngol.–Head Neck Surg..

[37] Ronconi G., Spagnolo A.G., Ferriero G., Giovannini S., Amabile E., Maccauro G., Ferrara P.E. (2017). Caso Clinico di Sindrome della Spalla Dolorosa Post Lesione Iatrogena del Nervo Accessorio Spinale in Seguito a Svuotamento Latero-Cervicale del Collo per Carcinoma della Lingua: Ruolo della Riabilitazione e Aspetti Etico-Deontologici. Ig. Sanità Pubblica.

[38] Ekici E., Yüzbaşıoğlu Ü., Özkeskin M., Özden F. (2025). Effects of Exercise on Head and Neck Cancer Surgery: A Systematic Review of Randomized Controlled Trials. Eur. Arch. Oto-Rhino-Laryngol..

